# Role and Importance of *Chlamydia Trachomatis* in Pregnant Patients

**DOI:** 10.3889/oamjms.2016.077

**Published:** 2016-08-05

**Authors:** Mariya Angelova, Emil Kovachev, Veselina Tsankova, Iliana Koleva, Silvia Mangarova

**Affiliations:** 1 *Department of Obstetrics and Gynecology, Trakia University, Faculty of Medicine, Stara Zagora, Bulgaria*; 2 *Department of Obstetrics and Gynecology, Paraskev Stoyanov Medical University, Varna, Bulgaria*

**Keywords:** *Chlamydia trachomatis*, pregnancy, neonatal infections, respiratory symptoms, polymerase chain reaction

## Abstract

**AIM:**

The aim of this study was to assess the prevalence of chlamydial infection among pregnant women and to determine the role of this infection in the fetus.

**MATERIAL AND METHODS:**

In the first phase of this study were reported 58 pregnant women with a positive test for active chlamydial infection by applying immunofluorescence. In the second phase of the study were reported pregnant with premature burst membranes (PBM), postnatal complications associated with chlamydial infection as puerperal endometritis, and newborns are monitored for low birth weight and growth retardation at birth.

**RESULTS:**

With a positive test are 58 patients in the first trimester or pregnancy registration in our consultation. After regimen with Sumamed (2 x 500 mg for three days and after 10 days again same scheme for them and their partner) at the beginning of the third trimester, the PCR test was made again. Of these, 5 were positive again, participants are between 20 and 30 years old. With premature rupture of OM are 20 patients. There was no increased incidence of premature births. Infants born to infected mothers have a higher risk of developing respiratory symptoms in the first 60 days of life. 3 of them have low for his age bodyweight.

**CONCLUSIONS:**

The scarcity of data on manifestations of chlamydial infection during pregnancy and neonatal outcomes justifies this study. Early diagnosis for registration of pregnancy and timely treatment of chlamydial infection as well as scrutinising the infection during the third trimester of pregnancy can prevent infection of the newborn. Therefore, preventive examinations should be considered as a priority for early detection of asymptomatic chlamydial infection in the conduct of antenatal care.

## Introduction

According to SZO genital chlamydiosis, today is considered the most common sexually transmissible infection. Various studies have been used for diagnosis of chlamydial infection in pregnant women; the frequency varies in different countries. Chlamydia Trachomatis during pregnancy can lead to miscarriage, premature birth, and premature rupture of membranes, intrauterine growth retardation, and puerperal endometritis [1-5].

Infection with *Chlamydia trachomatis* with the mother is associated with increased morbidity in newborns and infants up to three months. Approximately two-thirds of infants born vaginally to infected mothers will be infected at birth. These infections may lead to conjunctivitis, otitis media, pharyngitis and pneumonia in neonates. Moreover, neonatal infection with *Chlamydia trachomatis* may cause long-term consequences such as chronic obstructive pulmonary disease.

The scarcity of data on manifestations of chlamydial infection during pregnancy and neonatal outcomes justifies this study. Early diagnosis for registration of pregnancy and timely treatment of chlamydial infection as well as scrutinising the infection during the third trimester of pregnancy can prevent infection of the newborn [6]. Therefore, preventive examinations should be considered as a priority for early detection of asymptomatic chlamydial infection in the conduct of antenatal care.

The aim of this study was to assess the prevalence of chlamydial infection among pregnant women and to determine the role of this infection in the fetus, the results associated with miscarriage, premature birth, premature rupture of membranes and low birth weight.

## Materials and Methods

### 

#### Phase I

Endocervical samples were collected from 90 pregnant women. In the first phase of this study were reported 58 pregnant women with a positive test for active chlamydial infection by applying immunofluorescence.

#### Phase II study - maternal and perinatal data

In the second phase of the study were reported pregnant with premature rupture of membranes (PROM), postnatal complications associated with chlamydial infection as puerperal endometritis and infants were observed for low weight and hypotrophy at birth.

Pregnant women included in this study meet the following requirements for forming the record: information about age, marital status, the number of sexual partners in life and socio-economic status. Family income and level of education are the criteria used to determine the socio-economic status. Patients were excluded if the gestational age when less than 28 weeks and six days, having other infections that require antibiotic treatment or a history of use of antibiotics in the previous 30 days.

During the second stage of the study 45, pregnant women who meet the eligibility criteria were observed for 60 days after birth. From a total of 58 participants in the first phase of the study 13 were excluded from the study: four cannot be contacted and nine due to insufficient follow-up time (less than 40 days of birth).

A study was conducted in 45 infants who were evaluated within 24 hours and monitored thereafter every 15 days to 60 days of life. However, 10 newborns were excluded due to loss of contact.

## Results

### 

#### Phase I - study

Of 58 patients positive for the registration of pregnancy and treated with Sumamed (2 x 500 mg for three days and then 10 days later again the same scheme for them and their partner) at the beginning of the third trimester was made the immunofluorescence test again. Of these, 5 are positive again. The average age of the positive patients was 23.5 years with a standard deviation of ± 4.5 years.

Although we are looking for associations between *Chlamydia trachomatis* infection with family income (45.5%) and level of education (45.5%), no statistically significant relationship between them was found.

#### Phase II study - maternal and perinatal data

No statistically significant relationship between positivity for *Chlamydia trachomatous* infection and premature rupture of OM was found (Table 2). No correlation was seen for premature birth, and low birth weight (weight less than 2500 g) in chlamydia -positive women.

**Table 1:** Table for the presence of cervicitis or endocervical secretion in *Chlamydia trachomatis* positive patients

Vaginal examination	Chlamydia positive		Chlamydia negative	

Cervicitis	n	%	n	%
Yes No	2 3	3.45% 5.17%	4 49	6.90% 84.48%

Endocervical secretion				

Yes No	3 2	5.17% 3.45%	16 37	27.59% 63.79%

**Table 2:** Early rupture membranes in pregnant women with a positive test for *Chlamydia trachomatis*

Premature rupture of membranes(PROM)	Immunofluorescence test for *Chlamidia trachomatis*			

	Positive		Negative	

	n	%	n	%
Yes No Total	3 2 5	5.17% 3.45% 8.62%	17 36 53	29.31% 62.07% 91.38%

**Table 3:** Respiratory events in 58 infants born by mothers suffered *Chlamydia trachomatis*

At risk of Chlamydia trachomatis	Respiratory symptoms					

	Yes		No		Total	

	n	%	n	%	n	%
Yes	3	5.17%	2	3.45%	5	8.62%

No	5	8.62%	48	82.76%	53	91.38%

Total	8	13.89%	50	86.21%	58	100%

Caesarian section is recommended for chlamydia positive patients. During surgery and three days after it is recommended antibiotic, due to the risk of developing endometritis.

### Study of neonatal outcome

Of 58 newborns, 8 (13.79%), exhibit respiratory symptoms. Of these five infants (62.5%) were born of chlamydia -positive mothers. Moreover, infants born to infected mothers are more likely to develop respiratory symptoms such as nasal congestion, rhinitis, cough, and dyspnea.

## Discussion

Studies have shown that infection by *Chlamydia trachomatis* during pregnancy can lead to serious complications [7]. Prenatal screening still in the registration of pregnancy would be beneficial to reduce morbidity among patients themselves, but also as prevention of transmission to the newborn, and the partner.

The prevalence of *Chlamydia trachomatis* infection in this study was 8.62%. Although we found that the majority (45.5%) of patients with chlamydia -positive results had a low-income family, in addition, low level of education (45.5 percent) and were not aware of the existence of *Chlamydia trachomatis* and its impact on reproductive function. A total of 88% of positive on *Chlamydia trachomatis* in this study are married or live in cohabitation with their partner. Only one sexual partner was reported by 98% of women; 75% of chlamydia - positive patients were under 25 years of age. The risk of introduction of chlamydial infection is twice as high at 25 years young women. These findings indicate the need for sharing information about the risk factors for *Chlamydia trachomatis*. Given that 40 % of the chlamydia-positive patients are identified in cervicitis gynaecological examination, this is an important clinical finding, since in a subsequent pregnancy infection can ascend and lead to premature birth and neonatal infections.

Respiratory symptoms are more common in infants born by Chlamydia-infected mothers. It is, therefore, clear that the clinical findings in this study in infants born by Chlamydia positive pregnant women were associated with prenatal chlamydial infection of their mothers.

Our findings indicate the real need for prenatal screening for *Chlamydia trachomatis*. It is, therefore, necessary introduction and implementation of prenatal screening programs in laboratories and offices for outpatient care for detecting *Chlamydia Trachomatis*.
